# Ultrasound-Guided Intramural Inoculation of Orthotopic Bladder Cancer Xenografts: A Novel High-Precision Approach

**DOI:** 10.1371/journal.pone.0059536

**Published:** 2013-03-26

**Authors:** Wolfgang Jäger, Igor Moskalev, Claudia Janssen, Tetsutaro Hayashi, Shannon Awrey, Kilian M. Gust, Alan I. So, Kaixin Zhang, Ladan Fazli, Estelle Li, Joachim W. Thüroff, Dirk Lange, Peter C. Black

**Affiliations:** 1 The Vancouver Prostate Centre and Department of Urologic Sciences, University of British Columbia, Vancouver, BC, Canada; 2 Department of Urology, Johannes Gutenberg University, Mainz, Germany; 3 Department of Urology, Johann Wolfgang Goethe University, Frankfurt, Germany; University of California Irvine, United States of America

## Abstract

Orthotopic bladder cancer xenografts are essential for testing novel therapies and molecular manipulations of cell lines *in vivo*. Current xenografts rely on tumor cell inoculation by intravesical instillation or direct injection into the bladder wall. Instillation is limited by the lack of cell lines that are tumorigenic when delivered in this manner. The invasive model inflicts morbidity on the mice by the need for laparotomy and mobilization of the bladder. Furthermore this procedure is complex and time-consuming. Three bladder cancer cell lines (UM-UC1, UM-UC3, UM-UC13) were inoculated into 50 athymic nude mice by percutaneous injection under ultrasound guidance. PBS was first injected between the muscle wall and the mucosa to separate these layers, and tumor cells were subsequently injected into this space. Bioluminescence and ultrasound were used to monitor tumor growth. Contrast-enhanced ultrasound was used to study changes in tumor perfusion after systemic gemcitabine/cisplatin treatment. To demonstrate proof of principle that therapeutic agents can be injected into established xenografts under ultrasound guidance, oncolytic virus (VSV) was injected into UM-UC3 tumors. Xenograft tissue was harvested for immunohistochemistry after 23–37 days. Percutaneous injection of tumor cells into the bladder wall was performed efficiently (mean time: 5.7 min) and without complications in all 50 animals. Ultrasound and bioluminescence confirmed presence of tumor in the anterior bladder wall in all animals 3 days later. The average tumor volumes increased steadily over the study period. UM-UC13 tumors showed a marked decrease in volume and perfusion after chemotherapy. Immunohistochemical staining for VSV-G demonstrated virus uptake in all UM-UC3 tumors after intratumoral injection. We have developed a novel method for creating orthotopic bladder cancer xenograft in a minimally invasive fashion. In our hands this has replaced the traditional model requiring laparotomy, because this model is more time efficient, more precise and associated with less morbidity for the mice.

## Introduction

Bladder cancer is the fourth most common cancer in men and the ninth most common cancer in women in developed countries [1]. In 2010 there were 70,530 incident cases and 14,680 deaths from bladder cancer in the U.S. [2]. Approximately three quarters of cases are non-muscle invasive [3]. These have a high propensity for recurrence and a subset is at risk for progression to invasive disease [4]. The remaining one quarter of cases present as muscle invasive bladder cancer. Despite optimal multimodal therapy, approximately one half of patients with muscle invasive bladder cancer will succumb to their disease [5]. No significant breakthroughs have been made in the past two decades to enhance the systemic therapy of this patient population [6].

Murine models of human cancer using cell lines derived from patient tumors (xenografts) are an essential tool in cancer research. They allow us to interrogate tumor biology with molecular manipulation, to identify relevant diagnostic and predictive biomarkers, and to test antineoplastic effects of novel therapies. For bladder cancer, inoculation of human cell lines into the mouse bladder (orthotopic xenograft) is the reference standard [7], [8]. This inoculation can be achieved either by intravesical instillation of tumor cells (“intravesical model”) [9] or direct injection into the bladder wall (“intramural model”) [10]. Alternative models include the inoculation of murine bladder cancer cells in immunocompetent mice (syngeneic model) [11] as well as transgenic models [12]. Similar models are also possible in rats [13], [14].

Each orthoptic xenograft model has its shortcomings. Intravesical instillation leads to the formation of tumors on the urothelial surface of the bladder that are amenable to subsequent intravesical instillation of novel drugs. However, it has proven to be extraordinarily challenging using this method to achieve reliable tumor take with any cell lines other than KU7 [9], which we have recently demonstrated to be HeLa [15]. Furthermore, intravesical cell inoculation is time consuming and can lead to uncontrolled tumor growth in adjacent organs (urethra, ureter, renal pelvis) [16]. Finally, tumor location within the bladder is unpredictable, such that growth around the ureteral orifices can cause severe upper tract obstruction before mice reach therapeutic endpoints.

Direct injection of tumor cell into the bladder wall leads to the formation of invasive bladder tumors that are suitable for systemic treatments [10]. Although several cell lines grow reliably as xenografts in this model, the application is limited by the morbidity inflicted on the mice by the need for laparotomy and mobilization of the bladder [17]. It is also technically challenging to ensure adequate injection into the bladder wall, and this method is associated with a significant learning curve.

We have developed a novel approach to address these limitations of the intramural inoculation of bladder cancer xenografts, and thereby potentially enhance the accuracy and reproducibility of this model. We have optimized the percutaneous, ultrasound-guided injection of bladder cancer cells into the anterior bladder wall. In addition, we are able to monitor xenograft growth and perfusion *in vivo* longitudinally during therapy [18], [19], and we are able to inject therapeutic agents directly into the tumor under ultrasound guidance. Here we demonstrate the feasibility and reproducibility of ultrasound-guided intramural inoculation of orthotopic bladder cancer xenografts as well as subsequent image guided manipulation and monitoring.

## Materials and Methods

### Animals

Fifty 10-week-old female athymic nude mice were purchased from Harlan (Indianapolis, IN, USA). All animal procedures were performed according to the guidelines of the Canadian Council on Animal Care (CCAC). The protocol was approved by the Animal Care Committee of the University of British Columbia (Protocol Number: A10-0192).

### Tumor Cell Lines

The human bladder cancer cell lines UM-UC1, UM-UC3 and UM-UC13 were kindly provided by the Pathology Core of the Bladder Cancer SPORE at MD Anderson Cancer Center (Houston, TX, USA) [20]–[22]. Cell line identities were confirmed by DNA fingerprinting using the AmpFlSTR® Identifiler® Amplification protocol (Applied Biosystems, Carlsbad, CA, USA) [23]. All cell lines were cultured for less than 3 months in Dulbecco’s modified Eagle’s medium (DMEM) with 10% fetal bovine serum (FBS) at 37°C in a humidified 5% CO_2_ atmosphere.

For *in vivo* studies, the cell lines underwent transduction with a lentiviral construct carrying the luciferase firefly gene for *in vivo* imaging [9]. The luciferase plasmid contained a blasticidin resistance gene enabling positive selection with 10 µg/mL blasticidin (Invitrogen, Life Technologies Inc., Burlington, ON, Canada). Cell lines were controlled for in vitro luciferase activity and cell number was correlated with bioluminescence (R>0.99; data not shown), using the Xenogen IVIS Spectrum (Caliper Lifesciences, Hopkinton, MA, USA). For xenograft studies the cells were harvested at 70% confluence and suspended in Matrigel® (BD Biosciences, Mississauga, ON, Canada). The cell concentration was modified based on previously established growth kinetics of the three different cell lines (UM-UC1 luc 9×10^6^/mL; UM-UC3 luc 12×10^6^/mL; UM-UC13 luc 11×10^6^/mL).

### Percutaneous Tumor Inoculation

Tumor inoculation was performed with the Vevo 770® small animal imaging platform (Visual Sonics, Toronto, ON, Canada). A high frequency RMV 706 ultrasound scanhead (20–60 MHz), which allowed a lateral resolution of 30 micron and frame rates up to 240 fps, was used.

**Figure 1:**
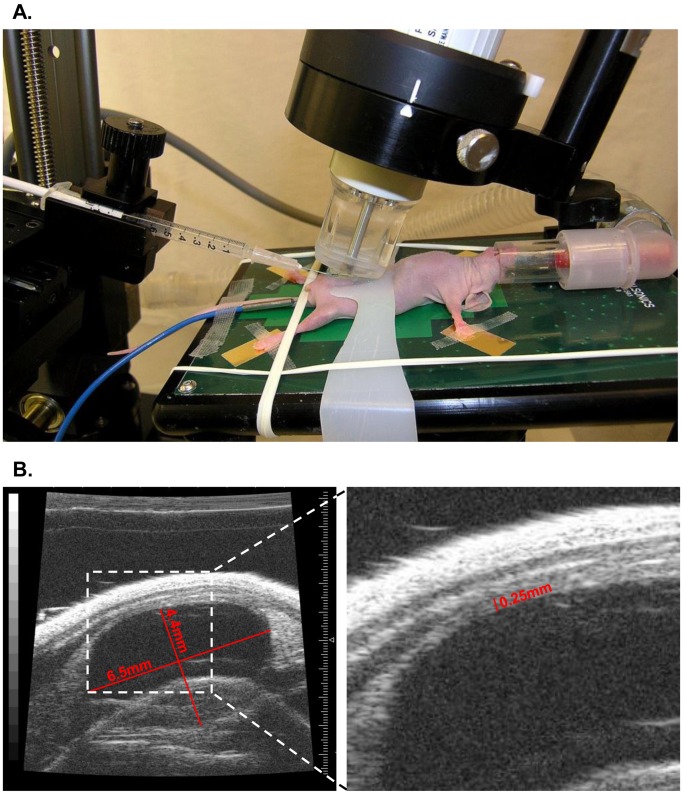
Ultrasound imaging. A. The mice were anesthetized with isoflurane and mounted on the heated imaging table with continuous monitoring of vital signs. After visualization of the bladder with the Vevo 700® small animal imaging platform the skin was perforated with a 30G needle. B. Ultrasound visualisation of normal mouse bladder in sagittal section with typical dimensions indicated (lumen dimensions 4.4×6.5 mm; wall thickness 0.25 mm).

The mice were anesthetized with isoflurane and mounted on the imaging table with continuous monitoring of vital signs [Fig. 1A]. The abdomen was disinfected with alcohol and sterile ultrasound gel was applied. The bladder was visualized on the screen [Fig. 1B] and the bladder lumen was filled with sterile, warm phosphate-buffered saline (PBS) through a 24 gauge angiocatheter to a desired volume of 50–100 µL.

**Figure 2:**
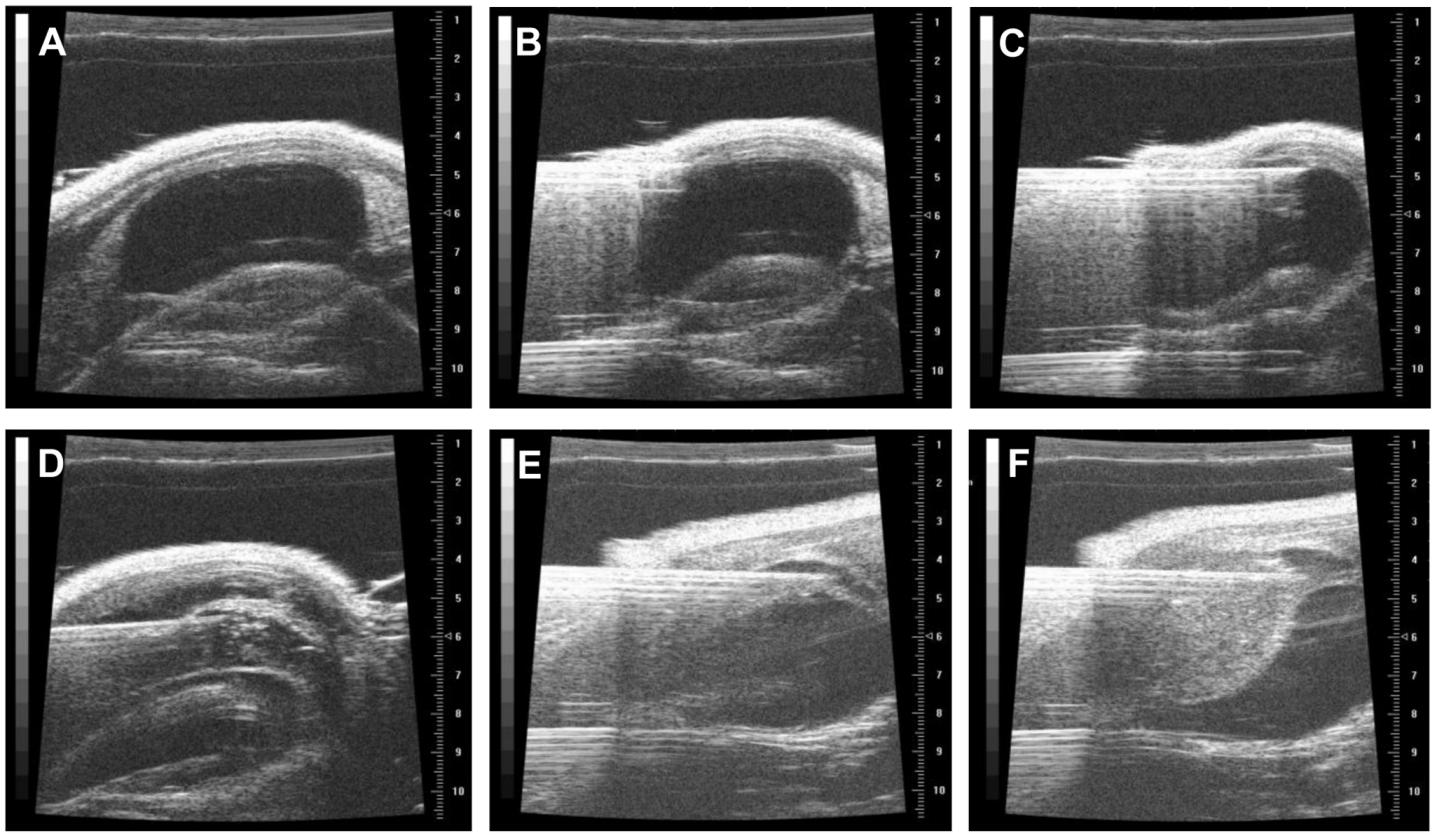
Inoculation of tumor cells. A. Detection of the needle on the ultrasound screen. B. Perforation of the skin and abdominal wall muscles. C. Needle insertion into the bladder wall without penetration of the mucosa. D. Injection of PBS (50 µl) between the muscular layer and the mucosa. E. Guidance of second needle into the artificially created space. F. Injection of tumor cells suspended in Matrigel®.

A 1.0 mL syringe filled with PBS and connected to a 30 gauge, ¾ inch needle (Kendall, Mansfield, MA, USA) was brought to the skin just above the pubic bone at an angle of 30° with the bevel directed anteriorly. After detection of the needle on the ultrasound screen [Fig. 2A], it was passed through the skin and the abdominal wall muscles [Fig. 2B]. The bevel of the needle was turned 180° (directed posteriorly) before the tip was inserted into the bladder wall without penetration of the mucosa [Fig. 2C]. PBS (50 µL) was injected between the muscular layer and the mucosa to create a space [Fig. 2D] and the needle was withdrawn. A second 1.0 mL syringe (filled with cancer cells suspended in Matrigel®, (BD Biosciences)) with a 30 gauge, ¾ inch needle was guided into the same space [Fig. 2E]. 40 µL (UM-UC1 luc) or 50 µL(UM-UC3 luc, UM-UC13 luc) of the cell suspension were injected into this space [Fig. 2F].

In two additional mice agarose was injected in a similar fashion in order to establish the exact location of xenograft inoculation within the bladder wall. The mice were immediately sacrificed and their bladders were removed for histologic analysis.

### Xenograft Growth Monitoring

Tumor growth was monitored by bioluminescence imaging (BLI) and ultrasound every third day starting 4 days after tumor inoculation. The Xenogen IVIS system was used for BLI and mice were imaged 10 and 15 minutes after intraperitoneal injection of D-Luciferin (150 mg/kg bodyweight; Firefly, Caliper Life Science). 3D ultrasound was performed in anesthetized mice with scanning of the bladder as a whole in 0.1 mm steps. The tumor volume was determined using the Visual Sonics imaging software package by analysis of every fifth picture according to the user manual [24].

### Microbubble Contrast-Enhanced Ultrasound Analysis of Tumors

To visualize the perfusion status of the xenograft tumors, a cine loop was recorded as the reference. A second cine loop (1000 frames at 30 Hz) was recorded 10 seconds after injection of 120 µL non-targeted microbubbles (Visual Sonics) into the tail vein of anesthetized mice. The point at which microbubbles entered the plane was determined and the background reference was subtracted. The tumor was selected as the contrast region and Reference Subtracted Mean Data were used. Changes of the Contrast Percent Area over time were documented and 2D images were recorded in which any pixel was marked green when a microbubble passed [24].

### Treatment

#### Intratumoral virus-injection

**Figure 3:**
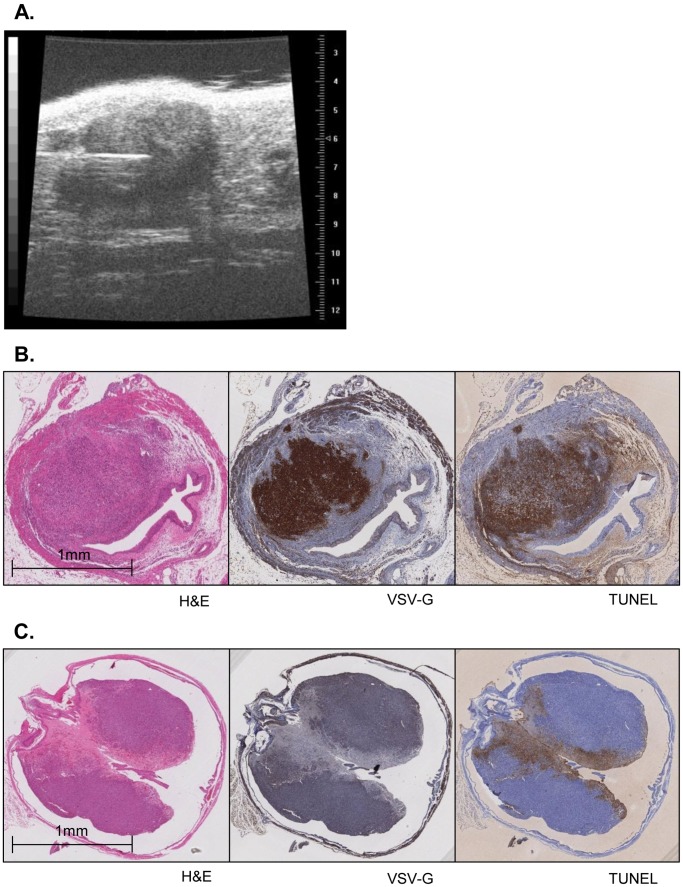
Ultrasound-guided intratumoral injection of treatment agents. A. The xenografts were visualized by ultrasound and either VSV (1.05×10^7^ pfu) dissolved in 25 µl PBS or PBS alone was injected through a 30G needle into the center of the tumor. B. 48 h after injection of VSV, all xenograft tumors showed positive staining for VSV-G around the injection site which correlated to TUNEL staining. C. VSV-G and TUNEL staining were negative after PBS injection alone.

To demonstrate the potential for ultrasound guided percutaneous intratumoral injection of treatment agents into established xenografts, we injected oncolytic virus (VSV) into UM-UC3 luc xenografts on day 22 after inoculation. Tumor burden as determined by BLI and ultrasound was used to divide the animals into two relatively equal groups. The tumors were visualized longitudinally by ultrasound and a 30G needle was inserted in the center of the tumor [Fig. 3A]. VSV (1.05×10^7^ pfu) suspended in 25 µL PBS was injected in 7 mice, and PBS alone was injected in the control group (n = 7).

#### Systemic chemotherapy

To demonstrate the potential for *in vivo* real-time monitoring of xenograft perfusion, we treated UM-UC13 luc tumor-bearing mice with cytotoxic chemotherapy. On day 28 after inoculation, the mice were divided into two equal groups based on tumor burden determined by ultrasound and BLI. Eight animals received intraperitoneal gemcitabine (120 mg/kg bodyweight; SANDOZ, Boucherville, QC, Canada) on day 30 and 35 as well as cisplatin (2.5 mg/kg bodyweight; Hospira, Saint-Laurent, QC, Canada) on day 31 and 36. An equivalent volume of PBS was injected at the same time points in the control animals (n = 7).

### Histology

UM-UC3 luc, UM-UC1 luc and UM-UC13 luc xenografts were harvested after 24, 28 and 37 days, respectively. At necropsy, the pelvis, retroperitoneum, liver and lungs were carefully inspected for possible metastasis, and any suspicious tissue was removed. This tissue and the whole bladders were fixed in formalin, embedded in paraffin and cut in 4-µm sections which were stained with haematoxylin and eosin (H&E). Depth of tumor invasion was determined by a pathologist (LF). For UM-UC3 tumors a T stage was applied according to 7^th^ edition of the American Joint Committee on Cancer/International Union Against Cancer (AJCC/UICC) TNM classification [25].

Detection of apoptotic cells by the TUNEL (Terminal deoxynucleotidyl transferase dUTP nick end labeling) technique was performed using Terminal transferase (#03333566001, Roche Applied Science, Indianapolis, IN, USA), dATP (D4788, Sigma-Aldrich, St. Louis, MO, USA) and DIG-11-dUTP (#1558706, Roche Applied Science). Using polyclonal rabbit antibody against the G protein of VSV (VSV-G; 1∶300, ab1874, Abcam, Cambridge, MA, USA) immunohistochemical staining was conducted by the Ventana autostainer model Discover XT (Ventana Medical System, Tuscon, AZ, USA) with an enzyme-labeled biotin streptavidin system and solvent-resistant 3,30-diaminobenyidine Map kit. All samples were subsequently analysed by a pathologist (LF) and the percentage of immunoexpression for VSV-G and staining for TUNEL was detected at 200× magnifications.

### Statistical Analysis

For statistical analyses, the mean bioluminescence and tumor volumes with their standard deviations were determined. The significance of differences was measured by Student’s t test (GraphPad Software Inc., San Diego, CA, USA) and P<0.05 was considered significant. Regression plots were used to describe the correlation between bioluminescence and volume.

## Results

### Tumor Cell Inoculation

**Figure 4:**
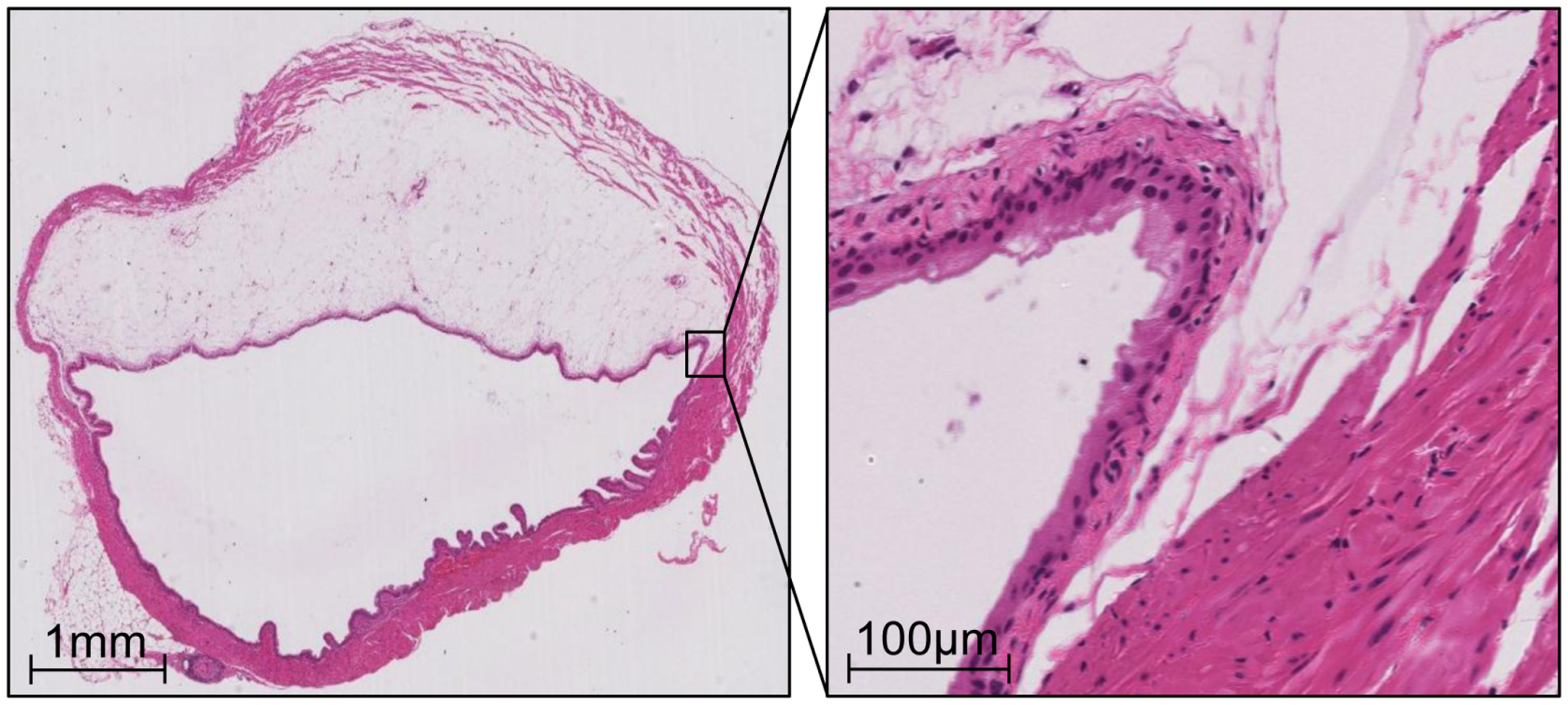
Intramural injection of agarose. The sagittal H&E section of a whole murine bladder demonstrates a layer of gel strictly in the lamina propria between mucosa and the muscularis propria.

Ultrasound-guided tumor cell inoculation was successfully performed in 50 animals (UM-UC1 luc 20 animals, UM-UC3 luc 15 animals and UM-UC13 luc 15 animals) [Table 1]. The typical experimental set-up and representative ultrasound images and dimensions of the murine bladder are depicted in Fig. 1. The steps of inoculation are shown in Fig. 2. The mean time per procedure (mounting of mice on the table until finished injection) could be decreased from 7.7±3.7 min for the first group (UM-UC3 luc) to 3.4±1.6 min for the third group (UM-UC1 luc). None of the animals suffered any complication during the procedure. Injection of agarose gel demonstrated that the inoculation was strictly located between the lamina propria and the tunica muscularis [Fig. 4].

**Table 1:** Percutaneous tumor cell injection – procedure, complications and growth.

*Inoculated cell line*		UM-UC1 luc	UM-UC3 luc	UM-UC13 luc
***Number of mice***		50		
		20	15	15
***Anesthesia***		Isoflurane	Isoflurane	Isoflurane
***Time per animal, min***		3.4 (+/−1.6)	7.7 (+/−3.7)	6.8 (+/−2.9)
***Anesthesia related deaths***		0	0	0
***Volume injected, µL***		40	50	50
***Cell count, absolute***		3.6×10^5^	6×10^5^	5.5×10^5^
***Tumor incidence***		49 (98%)		
		20 (100%)	14 (93%)	15 (100%)
***Extravesical tumor growth***		2 (10%)	–	–
***Intraperitoneal dissemination***		–	1 (7%)	–
***Lymph node metastasis***		–	3 (20%)	9 (60%)
***Follow up (days)***		28	22 [before treatment]	28 [before treatment]
***Tumor volume (µL)***	*day 4*	11.6 (±1.3)	12.5 (±1.7)	14.4 (±1.3)
	*end of follow up*	394.6 (±72.4)	288.7 (±66.1)	78.3 (±13.4)
***Tumor luminescence (Photons/sec)***	*day 4*	4.6×10^8^ (±9.4×10^7^)	2.0×10^8^ (±3.7×10^7^)	5.8×10^8^ (±1.3×10^8^)
	*end of follow up*	1.9×10^10^ (±4.0×10^9^)	1.4×10^10^ (±2.3×10^9^)	1.5×10^10^ (±1.9×10^9^)

### Xenograft Growth Monitoring

All 50 animals showed detectable tumor in the anterior bladder wall on ultrasound on the 4^th^ day after inoculation. The start and end tumor volumes are summarized in Table 1. After inoculation of UM-UC3 luc, one mouse showed intraperitoneal tumor dissemination and one tumor involuted after day 7. Similar patterns were seen with BLI. All mice had detectable luminescence on the 4^th^ day, and this increased steadily in all but one mouse. The overall tumor uptake rate was 98%. These findings were confirmed at the time of necropsy.

**Figure 5:**
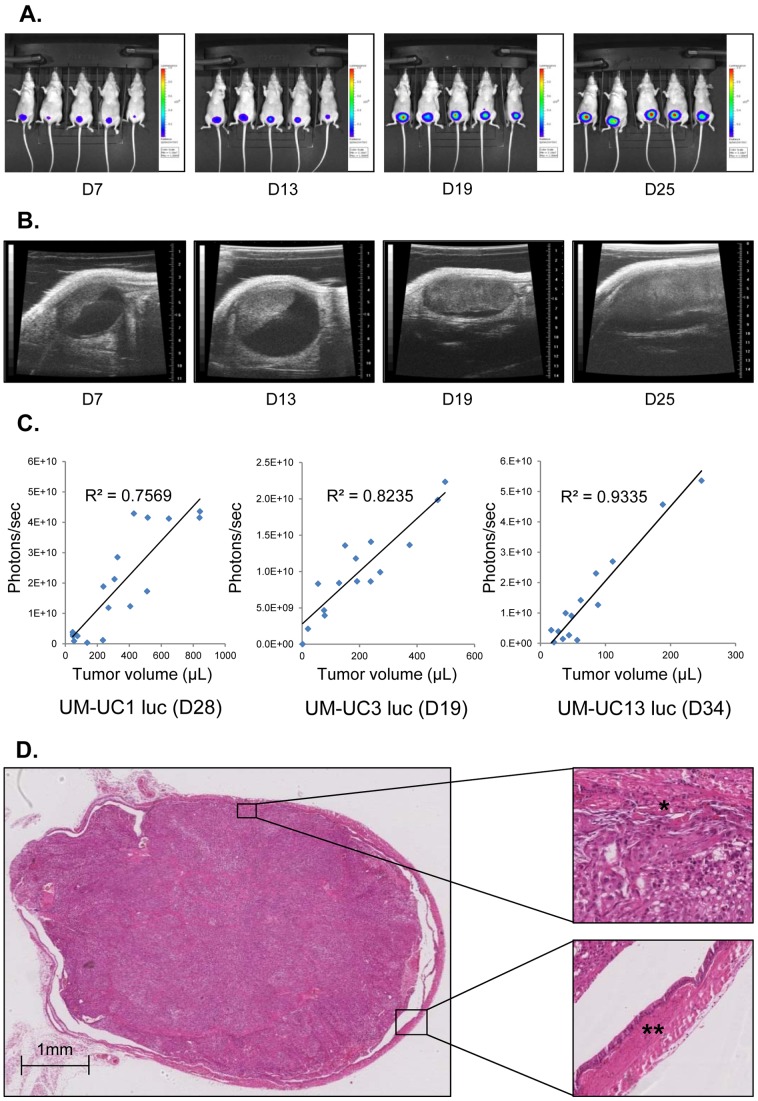
Longitudinal imaging of xenograft growth. Tumor growth was measured at regular time intervals by: A. bioluminescence imaging, and B. ultrasound. C. Correlation of bioluminescence and xenograft volume for all three cell lines. D. H&E section of a representative UM-UC1 luc xenograft demonstrating invasive growth into the muscle (*) without invasion into adjacent organs. All tumors originated from the anterior bladder wall and often occupied most of the bladder lumen without infiltrating the posterior wall (**).

During tumor growth [Fig. 5A, 5B] the ratio between entire tumor volume and BLI was not stable but variable depending on the time after tumor cell inoculation and the entire tumor volume itself. Although there had been a trend towards better correlation over the time course (R^2^ = 0.75, 0.82 and 0.92 for UM-UC1 luc at day 16, UM-UC3 luc at day 19 and UM-UC13 luc at day 34 respectively [Fig. 5C]), a drastic decline of the luminescence per mL tumor volume was noticed at high tumor volumes (data not shown).We have previously demonstrated a role for hypoxia and necrosis in this correlation [17].

Three mice (two UM-UC1 luc and one UM-UC3 luc) were sacrificed for humane reasons related to excessive tumor burden before the end of planned follow-up.

### Histology

Examination of the xenografts on H&E sections demonstrated that all tumors were invasive into muscle and some into perivesical fat, but there was no evidence of invasion into adjacent organs [Fig. 5D]. None of the tumors grew through the lamina propria into the bladder lumen. Retroperitoneal lymphadenopathy was noted in 60% of UM-UC13 luc and 20% of UM-UC3 luc xenografts. This was confirmed by H&E staining (data not shown). TNM staging was exemplary performed for all UM-UC3 luc tumors by histological analysis of the primary tumors and retroperitoneal lymph nodes, as well as macroscopic analysis of liver and lungs [Table 2].

**Table 2:** TNM Classification of UM-UC3 luc xenograft tumors.

		n	%
***T-Stage***	*pTa*	0	(0%)
	*pT1*	0	(0%)
	*pT2a*	5	(33%)
	*pT2b*	5	(33%)
	*pT3a*	4	(27%)
	*pT3b*	1	(7%)
	*pT4*	0	(0%)
***Lymph nodes***	*N+*	3	(20%)
	*N−*	12	(80%)
***Metastasis***	*M+*	1	(7%)
	*M−*	14	(93%)

After intratumoral injection of VSV into UM-UC3 luc xenografts [Fig. 3A], all 7 tumors showed positive staining for VSV-G. The same areas of the tumors staining for VSV-G also demonstrated staining in the TUNEL assay [Fig. 3B]. In contrast, VSV-G staining was negative for xenografts treated with PBS at the negative control [Fig. 3C].

### Response to Chemotherapy

**Figure 6:**
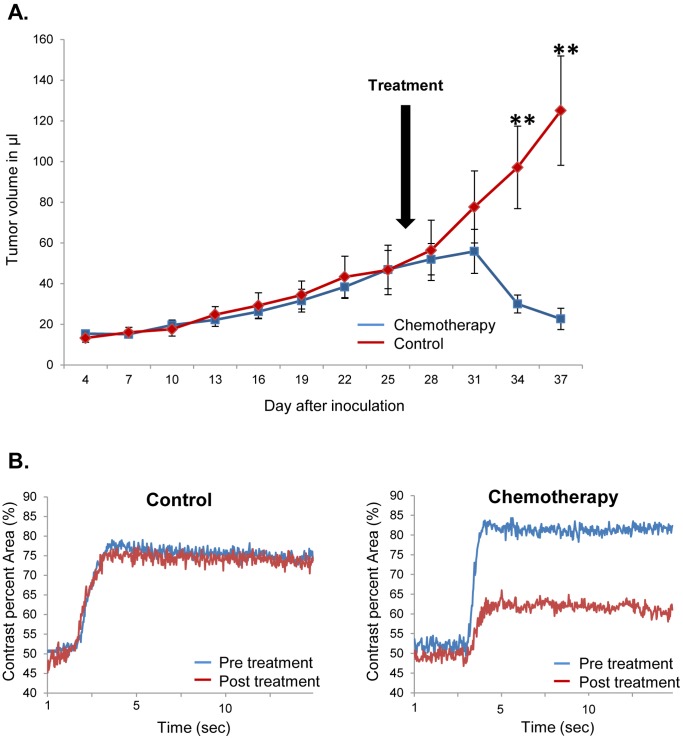
Treatment of xenograft tumors by chemotherapy. A. Mice bearing UM-UC13 luc tumors showed a remarkable decrease in tumor volume after systemic therapy with a combination of gemcitabine and cisplatin starting on day #28 after inoculation, compared to PBS control (** = P<0.01). B. Xenograft perfusion was measured by injection and ultrasound imaging of non-targeted microbubbles in UM-UC13 luc xenografts before and 5 days after administration of control agent (PBS; left panel) or systemic chemotherapy (gemcitabine/cisplatin; right panel). Perfusion was quantified as contrast percent area. Representative single results out of 4 measured animals per group are shown.

The mice bearing UM-UC13 luc tumors showed a remarkable response to the combination of gemcitabine and cisplatin. The median tumor volume significantly decreased from 48.6 µL (±7.7) to 25.3 µL (±5.8) after 7 days of therapy, whereas it increased from 48.8 µL (±14.8) to 114.5 µL (±26.8) in the control group [Fig. 6A].

The perfusion of the xenograft tumor was measured before and after administration of systemic chemotherapy with non-specific microbubbles. Chemotherapy led to a decrease in the perfusion rate (Contrast Percent Area) from 84% to 66%, whereas the same parameter remained constant after treatment with PBS alone (79% vs. 77%) [Fig. 6B].

## Discussion

The existence of reliable animal models is a basic requirement in oncologic research for the in vivo investigation of tumor biology and the testing of novel antineoplastic treatment strategies. Despite the existence of reproducible syngeneic [11] and transgenic [12] orthotopic tumor models of bladder cancer, they are not widely used due to both inherent limitations (e.g. questionable applicability of syngeneic models of murine bladder cancer to human disease) and complexity of the models, as well as the intensity of associated resource utilization. Orthotopic xenograft models have proven to offer the most flexibility (in terms of selection of cell lines) and have the most practical utility, and therefore remain the gold standard for in vivo modeling of bladder cancer [7], [8].

In this work, we have generated a novel in vivo model of orthotopic bladder cancer xenografts via the inoculation of human bladder cancer cells into the murine bladder and have shown it to be highly reproducible. Tumors were established in 98% of inoculated mice using three different human cell lines. Due to excellent optical resolution, the tumor cells can be inoculated by high precision strictly into the anterior bladder wall, thus reducing the rate of obstructive complications and allowing longer growth and treatment periods. Furthermore, the time per inoculation is short in comparison to existing models, and decreases rapidly with additional experience. The single observed complication was an intraperitoneal tumor cell dissemination which occurred in one of the first animals injected and can be attributed to the injection of too large a volume relative to the size of the animal. This was supported by the fact that reducing the volume of injection from 50 to 40 µL resulted in no further complications.

Our model is a modification of the orthotopic model previously described by Dinney et al. [10]. We believe that ultrasound-guided tumor inoculation enhances this model due to its ease, rapidity, accuracy and decreased degree of invasiveness. The latter factor is not only one of animal welfare, but may also contribute to reproducibility of experiments by decreasing confounding surgical complications. The accuracy of the standard intramural injection through a laparotomy is limited by the ability to determine exact needle placement at the time of injection. The shape and distribution of the bleb in the bladder wall after injection will often confirm correct location, but there is no a priori confirmation before the cells are injected. This means that a certain proportion of mice will have cells injected into the lumen of the bladder or spilled on the serosal surface of the bladder. With the ultrasound technique, a space is created submucosally in the bladder wall with saline, which carries no risk of spillage, and the subsequent tumor cell inoculation easily follows into the same space under direct visualization.

Monitoring tumor volume by ultrasound augments the information gained by BLI in the orthotopic xenograft model. While BLI has become an integral component of tumor detection and growth analysis, it does not always correlate well with tumor volume. We have previously shown in the orthotopic xenograft model that tumor perfusion and hypoxia confound the relationship between volume and luminescence [17], and the same is presumably responsible for the disparities shown here. This is particularly true in larger tumors [26], whereas early after inoculation ultrasound size may be inaccurate due to the volume of injected fluid, the surrounding tissue edema, and the fact that only a proportion of injected cells survive and grow.

An additional advantage of the ultrasound is the ability to inject novel treatment agents directly into the tumor. Here we have demonstrated the feasibility of intratumoral delivery of oncolytic VSV. The same technique would be amenable to other treatment strategies for bladder cancer such as gene therapy or the application of nanoparticles [27], [28]. As an example of the relevance of intratumoral injection in the treatment of human cancers, the intratumoral injection of DNA plasmid has recently been tested in the therapy of unresectable pancreatic cancers [29].

We have also demonstrated the ability to monitor tumor vascularity during drug treatment, in this case traditional cytotoxic chemotherapy. This ability will be particularly useful when evaluating the response of tumors to novel therapeutics including the development of resistance which may be reflected by a failure to diminish vascularity.

The principal limitation of ultrasound-guided tumor inoculation is the dependence on the ultrasound imaging platform, which may not be readily accessible to many researchers. Furthermore, this model requires familiarity with ultrasound imaging. On the other hand, complex animal modeling of human cancers, just like complex surgery on human patients, may best be performed by centers of excellence through scientific collaborations.

Our model does not necessarily substitute for the intravesical instillation of bladder cancer cell lines [9], [30]. The intramural model is superior to the intravesical model for most applications because of the ability to use multiple different cell lines. The intramural model, however, has no utility for testing novel therapies delivered intravesically because the tumors do not disrupt the urothelial surface (lamina propria) in the bladder lumen, and drugs therefore do not easily penetrate into the tumor. These tumors grow expansively away from the lumen of the bladder so that drugs administered into the lumen of the bladder cannot penetrate the full depth of the tumor. Furthermore, the intramural model represents muscle invasive disease, which is never treated with intravesical therapy in clinical practice. In the intravesical tumor model, on the other hand, the tumor surface is exposed in the bladder lumen and the tumor generally does not grow beyond the lamina propria for several weeks [9], so that drugs administered into the bladder lumen can adequately penetrate the tumor. The most significant limitation to the intravescial model, however, is its restriction to very few cell lines which, even in the most experienced hands, grow in only an unreliable fashion.

### Conclusions

We have successfully developed a technique for ultrasound-guided inoculation of orthotopic bladder cancer xenografts that significantly enhances pre-existing models of bladder cancer. The major advantages of this model lie in the rapidity and ease of tumor inoculation as well as in the accuracy and reproducibility of the model. Furthermore, we are able to monitor tumor volume longitudinally with ultrasound, to measure in vivo tumor perfusion with microbubble contrast agents, and to inject therapeutic agents into the tumor under ultrasound guidance.
